# The RENAPE observational registry: rationale and framework of the rare peritoneal tumors French patient registry

**DOI:** 10.1186/s13023-017-0571-y

**Published:** 2017-02-17

**Authors:** L. Villeneuve, G. Passot, O. Glehen, S. Isaac, F. Bibeau, P. Rousset, F. N. Gilly, on behalf of the RENAPE Network, Julio Abba, Karine Abboud, Mohammad Alyami, Catherine Arvieux, Gerlinde Averous, Naoual Bakrin, Gisèle Balagué, Vincent Barrau, Houda Ben Rejeb, Jean-Marc Bereder, Isabelle Berton-Rigaud, Isabelle Bonnefoy, Dominique Bouzard, Ivan Bricault, Cécile Brigand, Sébastien Carrère, Cécile de Chaisemartin, Madleen Chassang, Anne Chevallier, Thomas Courvoisier, Peggy Dartigues, Anthony Dohan, Clarisse Dromain, Julien Dubreuil, Frédéric Dumont, Clarisse Eveno, Marie Faruch-Bilfeld, Gwenaël Ferron, Juliette Fontaine, Laure Fournier, Johan Gagniere, Delphine Geffroy, Laurent Ghouti, Laurence Gladieff, Diane Goéré, Aymeric Guibal, Jean-Marc Guilloit, Frédéric Guyon, Bruno Heyd, Christine Hoeffel, Constance Hordonneau, Peggy Jourdan-Enfer, Rachid Kaci, Reza Kianmanesh, Catherine Labbé-Devilliers, Joëlle Lacroix, Bernard Lelong, Agnès Leroux-Broussier, Yoann Lherm, Réa Lo Dico, Gérard Lorimier, Caroline Malhaire, Frédéric Marchal, Pascale Mariani, Emilie Mathiotte, Pierre Meeus, Eliane Mery, Simon Msika, Cédric Nadeau, Pablo Ortega-Deballon, Olivier Pellet, Patrice Peyrat, Nicolas Pirro, Marc Pocard, Flora Poizat, Jack Porcheron, Anaïs Poulet, François Quenet, Patrick Rat, Pierre Rousselot, Hélène Senellart, Martine Serrano, Vincent Servois, Olivia Sgabura, Andrea Skanjeti, Magali Svrcek, Raphaël Tetreau, Emilie Thibaudeau, Yann Touchefeu, Jean-Jacques Tuech, Séverine Valmary-Degano, Delphine Vaudoyer, Stéphane Velasco, Véronique Verriele-Beurrier, Romuald Wernert, Franck Zinzindohoue

**Affiliations:** 1 Hospices Civils de Lyon, Pôle Information Médicale Evaluation Recherche, Unité de Recherche Clinique, Lyon, France; 2 EMR 3738, Lyon 1 University, Lyon, France; 3 Department of Digestive Surgery, Centre Hospitalier Lyon Sud, Pierre-Bénite, France; 4 Department of Pathology, Centre Hospitalier Lyon Sud, Pierre-Bénite, France; 5 Department of Pathology, Centre Hospitalier Universitaire, Caen, France; 6 Department of Radiology, Centre Hospitalier Lyon Sud, Pierre-Bénite, France; 7 RENAPE, Hospices Civils de Lyon, Centre Hospitalier Lyon Sud, 165 Chemin du Grand Revoyet, 69495 Pierre-Bénite, France

**Keywords:** Rare cancer network, Patient registry, Rare peritoneal tumor, Pseudomyxoma peritonei, Peritoneal mesothelioma

## Abstract

**Background:**

Rare peritoneal cancers represent complex clinical situations requiring a specific and multidisciplinary management. Because of their rarity, lack of awareness and knowledge often leads to diagnostic delays and misdiagnosis. And patients are not systematically referred to expert centers as they should be. Clinicians and researchers also face unique challenges with these rare cancers, because it is hard to conduct adequately powered, controlled trials in such small patient population. This is how an observational patient registry constitutes a key instrument for the development of epidemiological and clinical research in the field of these rare cancers. It is the appropriate tool to pool scarce data for epidemiological research and to assess the impact of diagnostic and therapeutic strategies. We aimed to provide the outlines and the framework of the RENAPE observational registry and share our experience in the establishment of a national patient registry.

**Results:**

The RENAPE observational registry has been launched in 2010 thanks to institutional supports. It concerns only patients with a histological diagnosis confirming a peritoneal surface malignancy. A web secured clinical database has been implemented based on data management procedures according to the principles of international recommendations and regulatory statements. A virtual tumor bank is linked in order to the conduct translational studies. Specialized working groups have been established to continuously upgrade and evolve the common clinical and histological data elements following the last classifications and clinical practices. They contribute also to standardize clinical assessment and homogenize practices.

**Conclusions:**

The RENAPE Registry may improve awareness and understanding of the rare peritoneal tumors into the incidence, prevalence, recurrence, survival and mortality rates, as well as treatment practices thereby enabling therapeutic intervention to be evaluated and ultimately optimized.

**Trial registration:**

ClinicalTrials.gov Identifier: NCT02834169

## Background

Peritoneal carcinomatosis most commonly represents local or regional evolution of an abdominal carcinoma. Sometimes it can be synchronous with the primary tumor (primary carcinomatosis) but more often is present as recurrent disease (metachronous or secondary) after first-line treatment of the primary tumor. When peritoneal masses are discovered, the principal diagnostic concern is metastatic disease, which is the most frequently encountered neoplastic process that involves the peritoneal cavity. However, primary peritoneal tumors should be appropriately included in the differential diagnosis in patients presenting with diffuse or focal peritoneal disease processes. Primary peritoneal tumors are an uncommon group of diverse pathologic disorders that share a common anatomic site of origin and have overlapping imaging features, yet are distinctly different clinically. Tumors originating from the peritoneum itself are definitely rarer and represent complex clinical situations requiring a specific management in expert centers. Differentiating primary peritoneal tumors from metastatic disease is important clinically so that patient management is appropriate. An epidemiological surveillance based on a specific tool is needed to better understand their characteristics and evolve the standards of care.

### Pseudomyxoma peritonei

Pseudomyxoma peritonei (PMP) is an uncommon clinical entity with an estimated incidence of one to two per million per year worldwide. According to national data based on population, the annual incidence of PMP is 1,500 cases in the United States of America (USA) and approximately 27 cases or 1.7 to 2 per million per year in the Netherlands. The incidence in Asia is about one per million per year and is presumed to be about a quarter of that in USA [1]. The occurrence of PMP is slightly higher in women than in men [2–4]. PMP occurs in approximately two of every 10,000 laparotomies and is more common in women with an average age of 53 years [2]. PMP is characterized by a gelatinous ascite associated with mucinous tumor deposits spreading on peritoneal surface and potentially invading abdominal organs [5]. PMP generally originates from a perforated appendiceal tumor. The biology of the disease is poorly understood and no overall consensus exists on histopathological classification, although tumors are commonly classified in a binary classification into Low grade appendiceal mucinous neoplasm (LAMN) or high grade mucinous adenocarcinoma [6–8]. The current standard of care with curative intent involves a combination of complete cytoreductive surgery (CRS) with hyperthermic intraperitoneal chemotherapy (HIPEC) [9, 10]. The treatment strategy is complex, associated with significant morbidity and mortality and a substantial institutional, and surgical “learning curve” [11, 12]. For selected patients, it results in long term survival and cure [9, 13–15].

### Peritoneal mesothelioma

Peritoneal Mesothelioma (PM) is a rare malignancy [16]. The incidence rates (per 1,000,000) range between 0.5 and three cases in men and between 0.2 and two cases in women [16]. A review of non-pleural mesotheliomas using data from surveillance, epidemiology and end results (SEER) estimated that mesothelioma numbers are about 2,500 per year in USA, with peritoneal being about 10–20% [17]. There is a predominance in the male population with a sex ratio of 3:1 (male:female) [16, 18]. The median age at diagnosis ranges from 49 to 55.7 years [19]. Occupational and environmental asbestos exposure seems to be causative in some cases of PM [20, 21]. Other even less frequent associations have been reported as implicated factors favoring the development of the PM as radiation therapy, SV 40 virus, chronic peritonitis, thorium dioxide or mica exposure [19, 22–25]. PM is locally aggressive neoplasm that comprises low-grade variants, such as multicystic and papillary well-differentiated mesothelioma, and highly malignant counterparts [26]. PM is confıned to the serosal surface of the peritoneal cavity from the mesothelial cells overlying peritoneum. It has long been considered a preterminal condition amenable only to palliative treatment with a historical median survival of less than 12 months [22]. Over the past decades, the combined approach of extensive CRS and HIPEC has emerged as a therapeutic modality for this disease and is admitted as the standard of treatment offering the longest survival and cure [27–33].

### Primary peritoneal serous carcinoma

Primary peritoneal serous carcinoma (PPSC) is a rare condition compared to their ovarian counterpart (6 vs. 120 women cases per million respectively) [34]. PPSC is an extra-ovarian primary peritoneal malignancy, histologically identical and clinically similar to advanced stage serous ovarian carcinoma. PPSC can occur many years after ovary removal surgery performed for benign diseases or prophylactic oophorectomy. The tumor appears during adulthood with a median age at diagnosis of 62 years [35, 36]. Women with breast cancer type 1 (BRCA1) gene mutations present an increased risk of developing a PPSC [35]. The therapeutic approach combining CRS with HIPEC has recently demonstrated a survival benefit in patients with PPSC when compared to those treated with surgery alone or surgery in combination with systemic chemotherapy [37].

### Peritoneal desmoplastic small round cell tumors

Desmoplastic small round cell tumor (DSRCT) is a rare abdominal tumor but highly fatal malignancy with only approximately 450 cases described in the literature (among which more than 60 are case reports) since its first description in 1991 by Gerald et al. [38, 39]. No large population data exists regarding the epidemiology of this tumor due to its rarity. Previous studies reported that DSRCT was found to be more prevalent in males [40]. This tumor type has a strong tendency to spread within the peritoneum but can also give rise to extraperitoneal metastases, mainly in the liver and lungs [39, 41]. A unique chromosomal translocation t(11;32)(p13;q12), of the Ewing sarcoma gene breakpoint region 1 (EWSR1) on 22q13 and the Wilms tumor gene (WT1) on chromosome 11p13 is highly specific and allows a formal diagnosis of DSRCT [42]. Without large series in the literature, standard of care for treatment of DSRCT remains unclear and challenging. However a multimodality approach with chemotherapy, surgery and radiotherapy appears to represent optimal management [39].

### Diffuse peritoneal leiomyomatosis

Diffuse peritoneal leiomyomatosis (DPL) is rare disease. Fewer than 150 cases have been reported in the literature to date. DPL is characterized by the proliferation of multiple benign smooth muscle cell-containing nodules in the peritoneal cavity. DPL manifests during adulthood and is predominantly found in women. Only one case of DPL has been reported in a male [43]. Malignant transformation is rare and in a few cases [44]. Etiology is unknown but DPL seems to be a multifactorial disease with a genetic or hormonal component leading to metaplasia of peritoneal mesenchymal cells [45, 46]. Depending on the extent of the disease, first-line treatment for DPL is surgical excision or CRS [47].

The establishment of a the French network for rare peritoneal tumors (RENAPE) reference networks with a clinical database linked to a virtual tissue bank aims to improve outcomes and make easier the exchange of experience, information, data and best practices on rare peritoneal malignancies (RPM) amongst all stakeholders [48]. Because of establishing a patient registry is a complex process which requires a range of technical and organizational skills; this paper aims to provide a framework for the implementation of a patient registry in the field of RPM and includes the important aspects that need attention during this process.

## Methods

### Objectives and scope

By definition, a registry is “an organized system that uses observational study methods to collect uniform data (clinical and others) from individual patient to evaluate specified outcomes for a population defined by a particular disease, condition or exposure, and that serves one or more predetermined scientific, clinical, or policy purposes” [49]. The RENAPE Registry has been launched in 2010. It aims to monitor more prevalence and incidence of RPM in France, to establish natural history of these rare cancers. It is also intended to assess the clinical effectiveness of new interventions, to measure the quality of care and to provide an inventory of patients to re-contact for participation in epidemiological studies, clinical trials or for health technology assessment to monitor real access to treatments.

### Institutional support

The RENAPE Observational Registry benefits the full support of the national patients association against RPM (AMARAPE) and is endorsed by French National Cancer Institute (INCa) as a global priority in the field of Rare Cancer. It is also partner of the European Platform for Rare Disease Registries (EPIRARE) co-funded by the European Commission within the European Union Program of Community Action in the field of Public Health.

### Ethical and legal statements

The RENAPE Observational Registry complies with applicable local regulations and with the ethical principles laid down in the Declaration of Helsinki (Fortaleza 2013). The RENAPE Observational Registry has been approved by the Advisory Committee for Data Processing in Health Research at the Research French Ministry (CCTIRS - n°10.257). The RENAPE Observational Registry has been registered with French Data Protection Authority (CNIL - no. DR-2010-297) in accordance with the French Law 78-17 dated January 6th, 1978 relating to data processing, files and personal freedom and privacy.

### Patient population

The RENAPE Observational Registry includes only patients with RPM such as peritoneal mesothelioma (ORPHA168811/168816), pseudomyxoma peritonei (ORPHA26790), primary peritoneal serous carcinoma (ORPHA168829), peritoneal desmoplastic small round cell tumors (ORPHA83469), diffuse peritoneal leiomyomatosis (ORPHA71274). All patients were required a confirmation of histological diagnosis as determined by an expert pathologist [48]. The RENAPE Observational Registry database concerns only persons whose usual place of residence is France.

### Registry design

The RENAPE Observational Registry is a retrospective, longitudinal patient registry that has been implemented through a secure, fully Web-based application (EOL^©^). It has been launched as an open-ended project without a pre-defined stopping point. The access to the registry application has currently been opened at all specialized French centers with expertise in the treatment of RPM [48].

### Data entry and management

For each new patient a minimal data set is required (Table 1) including identification, demographic information and etiology. The RENAPE Observational Registry employs internal record linkage to identify and eliminate duplicate patient and tumor records. Each entry in the registry has a unique identifier (alphanumeric code). Identification codes are stored in an independent and safe database according to the French Data Protection Act. Patients are written and oral informed that a pseudonymisation method replacing any identifying characteristics of registered personal data with a pseudonym (alphanumeric code) provides a limited protection for the identity of data subjects. However all aggregated data or results provided from the registered data are strictly anonymous. The clinical data capture will be updated appropriately based on each patient’s clinical management. Clinical data are directly collected in a standard format by the clinicians. A resource person – a clinical research assistant – with specialized knowledge in the clinical pathways of the RPM - is provided by the coordination of the RENAPE Registry in order to help sites on data entry process. The RENAPE Registry common data elements are organized into 9 categories: patient information, preoperative work-up, peroperative data and 90-day postoperative follow-up (Table 2) and long term follow-up. We use preferentially appropriate scores (Peritoneal Cancer Index - PCI, completeness of cytoreduction score - CC-score) and validated classifications (CTCAE). Clear, operational definitions of data elements and standard instructions were provided to collect data consistently. At each participating site, a local pathologist validates and records histological subtype and grade of tumor on extended data set available to capture pathological details (Table 3). Correlation edits check the compatibility of different data elements within a record. These verifications are done as data are loaded into the database and any records failing edits are rejected and returned to the participating sites for verification and/or correction. The data management procedures follow in accordance with the principles of Good Clinical Practices (GCP). The scope of these standard operating procedures is to: staff training and qualifications, case inclusion, case ascertainment, procedures for adding new cases to the permanent data set, rules for updating or changing data on file, follow-up, data exchange. Data quality is assured by pre-testing and consistency checks during data entry, when applicable. Data originate from various sources and specific attention is taken to avoid duplicate information.

**Table 1:** Minimum data set

Identification	
Surname	
Birth name	
Name	
Demographics	
Birth date	
Sex	
Etiology	
Peritoneal mesothelioma	
Pseudomyxoma peritonei	
Primary peritoneal serous carcinoma	
Peritoneal desmoplastic small round cell tumors	
Diffuse peritoneal leiomyomatosis

**Table 2:** Common clinical data elements

Patient information	
Contact details	
Current or past professional activity	
Medical contact details	
Personal and family relevant medical history	
Preoperative work	
Occupational/environmental exposure (asbestos, erionite, mica, etc.)	
Etiology	
Diagnosis date and circumstances	
Height	
Weight	
Imaging: MRI, CT-TDM, PET scan	
Biology: tumor markers, creatinine	
Neoadjuvant treatments:	
PIPAC	
Chemotherapy: dates, no. cycles, regimen	
Radiotherapy: dates, dose (Gy)	
Surgery: date, intervention (laparotomy, CRS)	
Peroperative data	
Date of surgery	
ASA score	
ECOG Performance Status	
Peritoneal Cancer Index (PCI)	
CCR score	
Spleen removal surgery	
Time of surgery	
Intraperitoneal chemotherapy:	
HIPEC modalities	
Chemotherapeutic agent(s) used	
Dose	
Type of postoperative analgesia	
90-day postoperative follow-up	
Major complications (based on CTCAE v4.0):	
Hematologic	
Cardiovascular	
Inflammatory fever	
Surgical	
Hemorrhage, hemoperitoneum, intra-abdominal hematomas	
Gastrointestinal	
Pulmonary	
Nephrologic	
Urologic	
Re-intervention (surgery)	
Image-guided drainage	
Endoscopic treatment	
Radiologic treatment	
Duration of ICU	
Hospital stay	
90-day vital status	

*Abbreviations*: *CCR* completeness of cytoreduction, *CRS* cytoreductive surgery, *CTCAE* Common Terminology Criteria for Adverse Events, *HIPEC* Hyperthermic Intraperitoneal Chemotherapy, *ICU* Intensive Care Unit, *PCI* Peritoneal Cancer Index, *PIPAC* pressurized intraperitoneal aerosol chemotherapy

**Table 3:** Histological data

General information	
Receive date	
Procedure: biopsy, resection	
Peritoneal cytology	
No. blocks	
Referent pathologist, surgeon	
RENA-PATH Working Group reviewing (if needed)	
Biobanking	
Patient consent form	
Conditions: frozen/formalin-fixed paraffin-embedded	
Biospecimen identification no./localization	
Pseudomyxoma peritonei	
Appendix diagnostic:	
No. blocks	
LAMN/adenocarcinoma	
Peritoneum diagnostic:	
No. blocks	
Signet ring cell	
Ronnett classification: DPAM, PMCA-I/D, PMCA	
WHO 2010 Classification: LAMN, high-grade mucinous adenocarcinoma	
Involved organs (list)	
Lymph node involvement: pNtot, pN+	
Peritoneal mesothelioma	
No. blocks	
Histologic forms:	
Epithelioïd	
Biphasic	
Sarcomatoïd	
Multicystic	
Well-differentiated papillary mesothelioma	
Lymph node involvement: pNtot, pN+	
Primary peritoneal serous carcinoma/peritoneal desmoplastic small round cell tumors/diffuse peritoneal leiomyomatosis	
Diagnostic	
No. blocks	
Lymph node involvement: pNtot, pN+	

*Abbreviations*: *DPAM* disseminated peritoneal adenomucinosis, *LAMN* low-grade appendiceal mucinous neoplasm, *PMCA* peritoneal mucinous carcinomatosis, *PMCA-I/D* peritoneal mucinous carcinomatosis with intermediate or discordant features, *pNtot* total number of dissected nodes, *pN+* number of involved nodes

### Data protection

The EOL^©^ application is validated under 21 Code of Federal Regulations criteria (CFR Part 11) [50]. Data protection is based on the definition of authorization profiles, defining the functions or types of information available to a user. The access to the electronic data capture application is controlled by personal username and password generated by the administrator. All recorded data must be validated by electronic signature. An audit trail module allows tracking of all accesses, modifications, and deletions of data. All exported files are archived with history within the system. The automatic identification of patients without nominative data enables an interoperability of the data recorded and remains pseudonymous. The RENAPE Registry benefits of a safe and secure hosting in France based on Information Technology Infrastructure Library (ITIL) security management (version 3).

### Follow up data

The RENAPE registry devotes substantial efforts to engage participating physicians in the registry to minimize and monitor loss to follow-up. If the patient has returned to the facility, records are obtained and appropriate information extracted. Physicians route the record to the RENAPE Registry coordination team. If the patient has not returned to the institution, follow-up letters are usually mailed to the managing or referring physician. Letters may be sent to other physicians involved in the care of the patient. A date of last contact should be requested in the letter for the patient or contact to complete (Table 4).

**Table 4:** Long term follow-up data

Date of last contact	
Vital status	
Date of death	
Primary cause of death	
Recurrence	
Date of recurrence	
Site(s) of recurrence: peritoneal, extraperitoneal	
Treatment modalities	
Surveillance	
Surgery: dates, intervention	
Intraperitoneal chemotherapy:	
Type do procedure: HIPEC, PIPAC	
Modalities, time	
Chemotherapeutic agent(s)used	
Dose	
Systemic chemotherapy: dates, no. cycles, regimen	
Radiotherapy: dates, dose (Gy)	
PIPAC: dates, regimen, doses	
Participation at a clinical trial	

*Abbreviation*: *PIPAC* pressurized intraperitoneal aerosol chemotherapy

## Results and discussion

Rare cancer patient registries as the RENAPE Registry represent a fundamental research tool for increasing knowledge on rare cancers by pooling data for fundamental and clinical research, epidemiological research. Patient registries and databases constitute key instruments for the development of clinical research in the field of rare cancers such RPM. They are the appropriate way to conduct research on populations and conditions that are not generally studied in clinical trials, yet are important for patients information and for clinical decision-makers [51] because they offer the opportunity to assess the feasibility of clinical trials, to facilitate the planning of appropriate clinical trials and to support the enrolment of patients. Because of its non-experimental design (i.e., no randomization), the present registry can be used to examine the impact of physician practice behaviors on quality of care.

The RENAPE Registry is based upon the voluntary participation of the centers, the percentage of institutions participating is high with all HIPEC French centers including international expert teams members of the Peritoneal Surface Oncology Group International (PSOGI). Unparticipating centers are usually low-volume centers, so that our data are likely skewed towards management practices that are mostly used in larger-volume centers. Therefore the RENAPE Registry is to be considered as a specialized patient-based registry. It doesn’t meet strictly the exhaustiveness criteria required for a nationwide population-based cancer registry. To optimize data quality and exhaustiveness, experienced clinical research resources help the centers to complete the data integrity. One of the main strength of the present registry is that detailed data are recorded to characterize patient clinical and pathological features, their multidisciplinary management and outcomes. Overall more than 500 fields have to be filled-in. The consistent longitudinal collection of patient data facilitates the creation of standards of care and dramatically improves patient outcomes even in the absence of new therapies [52]. Data collected also allow the impact therapeutic strategies and clinical practices to be assessed. Furthermore, the RENAPE registry benefits from the many advantages offered by the EOL^©^ application: privacy and security of the data; flexibility and scalability, simplicity and fast implementation. Therefore, the registry is always in evolution to stick to novel standard of care or classification. The experience and multidisciplinary of active contributors allow to continuously evolve this common tool and clinical, histological items from database are upgraded. The RENAPE network organization has gathered expert pathologists in the RENA-PATH specialized working group that participate at the process of defining consensus criteria and nomenclature for appendiceal tumors under the auspices of the PSOGI [7, 53]. The RENA-PATH Group also leads collaborative translational research projects based on the virtual biobank linked to the Registry clinical database [54, 55]. Biocollection is disseminated and locally stored in all associated biological resource centers (BSR). In translational collaborative studies, the RENAPE registry is used to screen patient population concerned and to provide survival data. Then analyses are only conducted on available biospecimens for which patients have given their local written consent. The RENA-RAD Working Group has been formed recently with expert radiologists in peritoneal carcinomatosis imaging [56]. They share experiences and develop common tools in order to standardize radiological assessment of patients who are suitable for CRS with HIPEC.

A multidisciplinary care management is required among clinicians, surgeons and experts from the RENAPE network in order to improve patient selection and optimize survival outcomes. Specialists use transversal tools for quantitative and qualitative assessment of patients who are suitable for surgical treatment. The PROMISE® internet application has been developed to allow for a standardized assessment of the peritoneal disease extent intended of multidisciplinary teams and centers that treat patients with RPM [56].

The RENAPE Registry’s implementation will clearly rise up to offer the opportunity to fill in important gaps in knowledge about RPM, through national and international collaborations [9, 33, 39, 53, 57, 58].

## Conclusions

The rarity of the RPM and their diagnostic uncertainties limit our understanding of its epidemiological features. There is therefore a clear need - recognized by specialists throughout France – for a specific RPM Observational Registry. The information collected from the RENAPE Registry will improve awareness and understanding of the RPM into the incidence, prevalence, recurrence, survival and mortality rates, as well as treatment practices thereby enabling therapeutic intervention to be evaluated and ultimately optimized. The Registry will also enable clinicians to review the prognosis of different patient groups and identify long-term therapeutic benefits of therapeutic interventions. The multi-institutional nature of the project and web-based database structure permit easy data entry and provide a mechanism to scale the RENAPE Registry to a larger consortium of contributing institutions.

## Abbreviations

AMARAPE: National patients association against rare peritoneal malignancies
BRCA1: Breast cancer gene one
BSR: Biological resource center
CC score: Completeness of cytoreduction score
CCTIRS: Advisory committee for data processing in health research
CFR: Code of federal regulations
CNIL: French data protection authority
CRS: Cytoreductive surgery
CTCAE: Common terminology criteria for adverse events
DPL: Diffuse peritoneal leiomyomatosis
DSRCT: Desmoplastic small round cell tumor
EOL: Electronic data capture solution designed by Medsharing^©^
EPIRARE: European platform for rare disease registries
ESWR1: Ewing sarcoma breakpoint region 1
GCP: Good clinical practices
HIPEC: Hyperthermic intraperitoneal chemotherapy
INCa: French National Cancer Institute
ITIL: Information technology infrastructure library
LAMN: Low grade appendiceal mucinous neoplasm
PCI: Peritoneal cancer index
PM: Peritoneal mesothelioma
PMP: Pseudomyxoma peritonei
PPSC: Primary peritoneal serous carcinoma
PROMISE: Peritoneal malignancy stage evaluation
PSOGI: Peritoneal surface oncology group international
RENAPE: French network for rare peritoneal tumors
RPM: Rare peritoneal malignancies
SEER: Surveillance, epidemiology and end results
SV: Simian virus
USA: United States of America
WT: Wild type
